# Self-Perceived Barriers to Pediatric Cancer Care in Sub-Saharan Africa: A Cross-Sectional Multinational Study

**DOI:** 10.1200/GO.24.00137

**Published:** 2025-02-06

**Authors:** Ole Stoeter, Nikolaus C.S. Mezger, Tamara Koenig, Eric Chokunonga, Girum Tessema, Adugna Fekadu Damise, Alda Stevy Makouanzi, Esther Majaliwa, Mahine Ivanga, Bakarou Kamate, Franck Gnahatin, Sarah Nambooze, Ima-Obong A. Ekanem, Toralf Bernig, Biying Liu, Sumit Gupta, Eva Johanna Kantelhardt

**Affiliations:** ^1^ Global and Planetary Health Working Group, Institute of Medical Epidemiology, Biometrics and Informatics, Martin-Luther-University Halle-Wittenberg, Halle, Germany; ^2^ Zimbabwe National Cancer Registry, Harare, Zimbabwe; ^3^ Department of Oncology, School of Medicine, Addis Ababa University, Addis Ababa, Ethiopia; ^4^ Addis Ababa University College of Health Sciences, Tikur Anbessa Specialized Hospital, Radiotherapy Center, Addis Ababa, Ethiopia; ^5^ Hôpital général Adolphe sicé, Brazzaville, Congo; ^6^ Kilimanjaro Christian Medical Center, Moshi, Tanzania; ^7^ Institut de Cancérologie d’Akanda, Libreville, Gabon; ^8^ Faculté de Médecine et d'Odonto-Stomatologie, Bamako, Mali; ^9^ Registre des cancers d'Abidjan, Programme National de lutte contre le Cancer, Abidjan, Côte d’Ivoire; ^10^ Kampala Cancer Registry, School of Biomedical Sciences, Makerere University, Makerere, Uganda; ^11^ Calabar Cancer Registry, Department of Pathology, University of Calabar Teaching Hospital, Calabar, Nigeria; ^12^ Department of Pediatrics, Martin-Luther-University Halle-Wittenberg, Halle, Germany; ^13^ African Cancer Registry Network, Oxford, United Kingdom; ^14^ Division of Hematology/Oncology, The Hospital for Sick Children, Toronto, Canada; ^15^ Faculty of Medicine, University of Toronto, Toronto, Canada; ^16^ Department of Gynaecology, Martin-Luther-University Halle-Wittenberg, Halle, Germany

## Abstract

**PURPOSE:**

The number of patients with childhood cancer (CC) in sub-Saharan Africa is expected to rise over the coming years. According to the WHO Initiative for Childhood Cancer, access to care is crucial and must be guided by the needs of patients and their families. Our study explored barriers to CC treatment from a patient's perspective to guide the health care providers.

**METHODS:**

From February to September 2021, we conducted a multinational cross-sectional study with a sample from nine population-based cancer registries in nine sub-Saharan countries. Inclusion criteria comprised a cancer diagnosis according to the International Classification of Childhood Cancer, age 0-19 years, and year of diagnosis 2017-2019. A questionnaire was administered asking families about self-perceived barriers accessing surgery, radiotherapy, and chemotherapy. To assess associated factors, we conducted a multivariable regression analysis presenting the results as odds ratios (ORs).

**RESULTS:**

A total of 224 patients with CC was included. The fear of treatment effects and the perceived (poor) health of the child were named most frequently as barriers for all treatment modalities (78.9% and 75.5%, respectively). For chemotherapy, respondents who indicated themselves as rich had lower odds of perceiving the (poor) health of the child as a barrier (OR, 0.06 [95% CI, 0.01 to 0.36]). For radiotherapy, long waiting time and (limited) availability in the country were more commonly barriers (OR, 7.53 [95% CI, 3.38 to 16.78]; OR, 11.11 [95% CI, 2.04 to 60.46], respectively) than for chemotherapy.

**CONCLUSION:**

Despite known barriers such as the availability of therapy, our study additionally indicates the importance of the patients' and families' perceptions of the disease and its treatment. Further expanding measures of social support for affected families should be regarded as one of the main pillars to assure access to care.

## INTRODUCTION

In 2030, 60,000 children (age 0-19 years) are expected to develop cancer in sub-Saharan Africa (SSA), a 20% increase from 2020.^1^ Several challenges remain to ensure quality childhood cancer (CC) care for patients in SSA, with survival rates considerably lower than in Europe and the United States.^2^ The WHO's Global Initiative for Childhood Cancer aims to increase the overall survival rate of patients with CC to at least 60% by 2030 globally.^3^ To allocate resources efficiently, organizing pediatric oncology services according to the patients’ and their caretakers’ needs is crucial. The International Pediatric Oncology Society (SIOP) created a framework for adapted treatment regimens in low- and middle-income countries (LMICs).^4^ This framework identifies logistical and social support for patients with CC and their families as basic measures, indicating that improvements in CC care are only sustainable if patients can effectively access treatment.

**CONTEXT**

**Key Objective**
To assess barriers from the patients' and families' perspective, which limit access to childhood cancer (CC) care in sub-Saharan Africa (SSA).
**Knowledge Generated**
The fear of treatment effects and the perceived poor health of the child were named most frequently as barriers accessing CC care. Barriers differed by sociodemographics, treatment modality, and geographic region.
**Relevance**
To increase access to care, social support and counseling for patients with CC and their families need to be an integral part of cancer control planning in SSA.

Previous research regarding needs of patients with CC and their caretakers mainly investigated reasons and risk factors for treatment abandonment, defined as the failure to start or complete the planned treatment.^5^ Only few studies focused on access to CC care in general, also considering barriers that did not lead to a discontinuation of treatment but may still delay the course of treatment and affect the (psychosocial) well-being of patients with CC and their families negatively. In a study from Kenya and Uganda, 82 guardians of patients with CC named restricted access to transport and household responsibilities as barriers to care.^6^

To provide guidance for CC care tailored to patients and their caretakers' needs, we assessed self-perceived barriers to CC care by performing telephone surveys in a population-based sample from SSA.

## METHODS

Between February and September 2021, we conducted a cross-sectional and multinational questionnaire study among patients and their families. Patients with CC diagnosed between July 2017 and December 2019 and age 0-19 years were included.

### Questionnaire Design

We developed a questionnaire comprising the following three domains: sociodemographic characteristics of the children and their main caretaker, information on diagnosis, and information on treatment and concomitant circumstances. Modality-specific barriers to surgery, radiotherapy, and chemotherapy were inquired using a three-point scale (unproblematic, problematic, very problematic). To categorize barriers and offer a comprehensive overview, we split the barriers according to the 5-A model of Penchansky et al into five groups: acceptability, availability, affordability, accommodation, and accessibility (Data Supplement).^7^ Patient referral implied whether patients and their families were aware of where to go to start treatment. The questions were only asked if the respective treatment modality was recommended, irrespective of sole recommendation, initiation, or completion. Treatment receipt was defined as initiation of the respective treatment modality, irrespective of treatment abandonment or completion. The questionnaire was designed in an interdisciplinary team of pediatric oncologists and Public Health experts, and based on literature review. The survey was piloted by phone interview among 40 patients with CC in Harare, Zimbabwe, Addis Ababa, Ethiopia, and (translated to French) in Brazzaville, Congo. After the pilot, few questions were altered for comprehensibility or removed. The final questionnaire took approximately 30 minutes. It contained a maximum of 115 questions depending on how many treatment modalities were received.

### Sample

**FIG 1:**
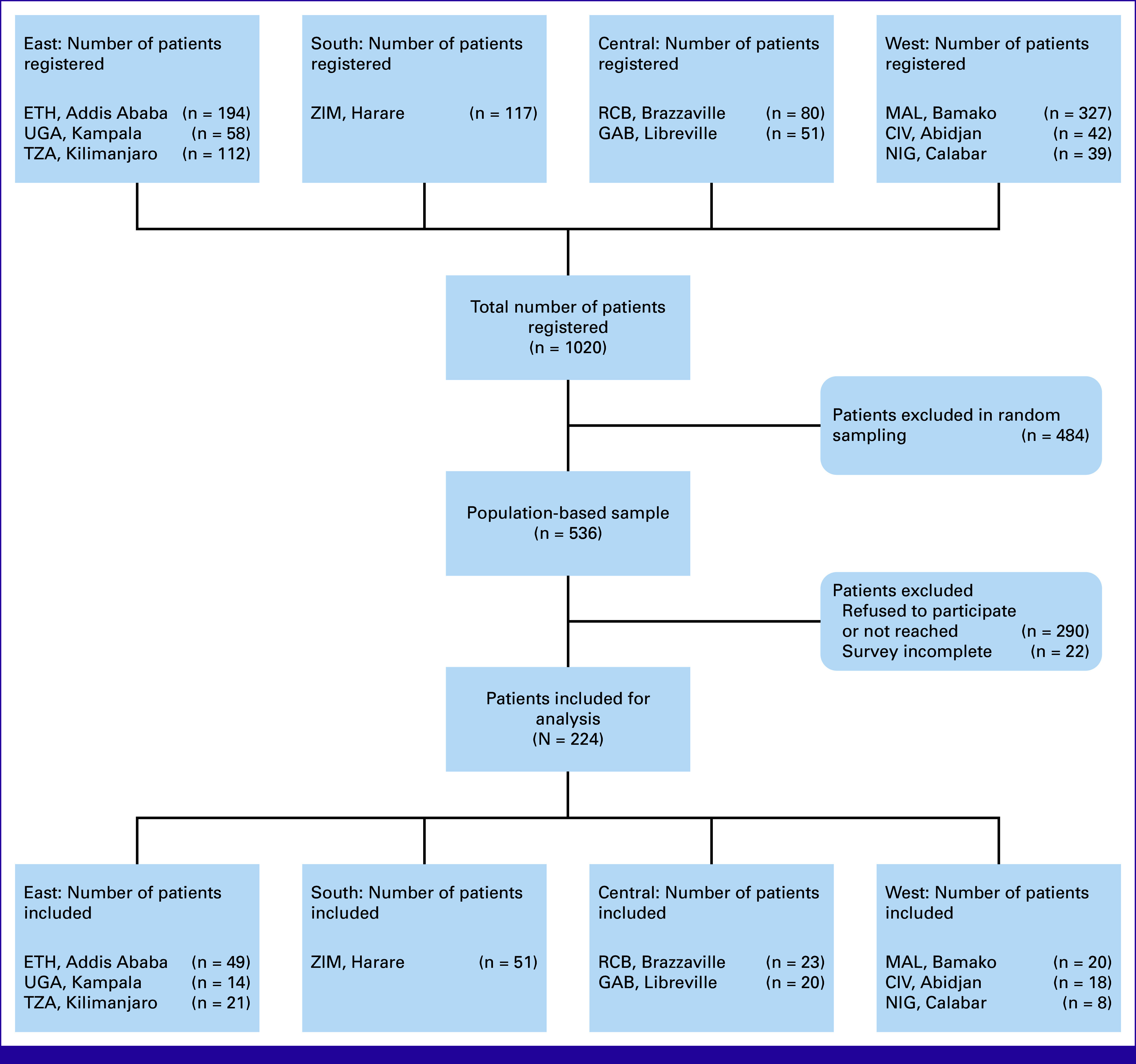
Flow chart. Number (n) indicates number of completed questionnaires per registry, and country codes indicate the location of the respective registry. CIV, Côte d‘Ivoire, NIG Nigeria; ETH, Ethiopia; GAB, Gabon; MAL, Mali; RCB, Republic of the Congo; TZA, Tanzania; UGA, Uganda; ZIM, Zimbabwe.

Nine population-based cancer registries (PBCRs) from all subregions of the African Cancer Registry Network (AFCRN) were selected as sites for data collection (Fig 1). Previously, all 31 AFCRN members were approached to ask for the feasibility of conducting the study. The selection process considered the availability of personnel and data and the regional allocation of the registries. The PBCRs co-operate with both public and private cancer facilities in respective geographic registry areas and register all patients newly diagnosed with cancer (AFCRN^8^).^9^

For Abidjan and Bamako, only patients with CC diagnosed in 2019 were included. Participation was voluntary and not incentivized. Age, sex, diagnosis, and date of diagnosis were abstracted from cancer registry records. Using contact information from these records, the survey was conducted via phone by health care professionals working at the registries or local hospitals. Data and verbal consent was documented in EpiData software.^10^ We intended to survey main caretakers when possible. Patients were only surveyed if they were age 15 years or older. In most cases, the main caretaker was a parent of the patient, and a small fraction of caretakers were direct relatives such as grandparents, aunts, or uncles. CC and its implications for the affected patients and families are sensitive topics. The questionnaire was voluntary and could be stopped at any point if requested by the respondents. The respondents were offered to contact the study team, for example, due to emotional distress.

### Statistical Analysis

RStudio Version 1.3.959 was used for analysis. According to the International Classification of Childhood Cancer, third edition, we subdivided CC into 12 diagnostic groups.^11^ For modality-specific factors contributing to (non)uptake of CC care, answers problematic and very problematic were merged as a barrier and stratified by modality and region. To assess the association of region with barriers to access to care, we conducted a multivariable regression analysis, using a generalized linear model, adjusting for age and sex of the patient, age and education of the caretaker, and self-perceived wealth. The multivariable analysis was planned to be conducted for each treatment modality separately. However, due to low patient numbers in surgery and radiotherapy, multivariable analysis was only possible for chemotherapy. Using an analysis of variance test, we tested whether there was a difference between a generalized linear and generalized linear mixed model. If so, the generalized linear mixed model was selected. As the regression models did not converge for all barriers, in some cases, the models were simplified by excluding region as a variable. The results of the multivariable regression analyses are presented as odds ratios (ORs). We compared analysis for patients with and without missing answers but did not find notable differences.

### Ethics

The study protocol was approved by the Research Committee of the AFCRN and by the Martin-Luther-University Halle-Wittenberg Review Board (votum 2020-192). Local ethical requirements were fulfilled by participating centers. The study protocol is in line with the Declaration of Helsinki. Results were reported according to the Strengthening the Reporting of Observational Studies in Epidemiology guidelines.

## RESULTS

Over the study period from July 2017 to December 2019 (18 months), the nine participating centers registered 1,020 patients with CC. Of those, 224 were included in our study, ranging from eight patients (Calabar) to 51 patients (Harare) per registry (Fig 1). We conducted an analysis comparing included versus excluded patients by age, sex, year of diagnosis, and diagnostic groups and found no major differences.

### Sociodemographic Characteristics

The median age of the patients with CC was 5 years. Concerning the patients' status at the time of the survey, the majority of respondents (52.7%) in all regions except Eastern Africa (45.2%) reported that the child had died. When asked about their self-perceived wealth, most caretakers reported to be middle class in Southern and Western Africa (78.4%; 58.7%), whereas in Eastern and Central Africa, most caretakers reported to be poor (51.2%; 69.8%; Table 1).

**TABLE 1:** Sociodemographic Characteristics of Patients and Caretakers by Region

Respondents' Characteristics	All, No. (%)	East, No. (%)	South, No. (%)	Central, No. (%)	West, No. (%)
Patient					
Age					
0-4	96 (42.9)	27 (32.1)	33 (64.7)	12 (27.9)	24 (52.2)
5-9	51 (22.8)	17 (20.2)	13 (25.5)	12 (27.9)	9 (19.6)
10-14	38 (17.0)	18 (21.4)	5 (9.8)	8 (18.6)	7 (15.2)
15-19	38 (17.0)	21 (25.0)	—	11 (25.6)	6 (13.0)
NA	1 (0.4)	1 (1.2)	—	—	—
Sex					
Male	130 (58.0)	49 (58.3)	32 (62.7)	24 (55.8)	25 (54.3)
Female	94 (42.0)	35 (41.7)	19 (37.3)	19 (44.2)	21 (45.7)
Year of diagnosis					
2017	7 (3.1)	5 (6.0)	—	2 (4.7)	—
2018	87 (38.8)	40 (47.6)	26 (51.0)	14 (32.6)	7 (15.2)
2019	130 (58.0)	39 (46.4)	25 (49.0)	27 (62.8)	39 (84.8)
Status					
Feeling healthy	78 (34.8)	35 (41.7)	20 (39.2)	12 (27.9)	11 (23.9)
Feeling sick	15 (6.7)	4 (4.8)	2 (3.9)	—	9 (19.6)
Child died	118 (52.7)	38 (45.2)	29 (56.9)	25 (58.1)	26 (56.5)
NA	13 (5.8)	7 (8.3)	—	6 (14.0)	—
Caretaker					
Age					
<30	34 (15.2)	11 (13.1)	11 (21.6)	5 (11.6)	7 (15.2)
30-39	91 (40.6)	29 (34.5)	34 (66.7)	13 (30.2)	15 (32.6)
40-49	63 (28.1)	25 (29.8)	4 (7.8)	19 (44.2)	15 (32.6)
≥50	29 (12.9)	17 (20.2)	2 (3.9)	4 (9.3)	6 (13.0)
NA	7 (3.1)	2 (2.4)	—	2 (4.7)	3 (6.5)
Religion					
Christian	179 (79.9)	71 (84.5)	51 (100)	35 (81.4)	22 (47.8)
Muslim	38 (17.0)	13 (15.5)	—	1 (2.3)	24 (52.2)
Other	3 (1.3)	—	—	3 (7.0)	—
No religion	3 (1.3)	—	—	3 (7.0)	—
NA	1 (0.4)	—	—	1 (2.3)	—
Number of children in the family					
1	33 (14.7)	12 (14.3)	9 (17.6)	2 (4.7)	10 (21.7)
2-3	113 (50.4)	46 (54.8)	29 (56.9)	19 (44.2)	19 (41.3)
4+	77 (34.4)	26 (31.0)	13 (25.5)	21 (48.8)	17 (37.0)
NA	1 (0.4)	—	—	1 (2.3)	—
Education					
Illiterate	18 (8.0)	8 (9.5)	—	—	10 (21.7)
Elementary	49 (21.9)	30 (35.7)	4 (7.8)	4 (9.3)	11 (23.9)
Secondary	87 (38.8)	24 (28.6)	36 (70.6)	16 (37.2)	11 (23.9)
University	69 (30.8)	22 (26.2)	11 (21.6)	23 (53.5)	13 (28.3)
NA	1 (0.4)	—	—	—	1 (2.2)
Self-perceived wealth					
Poor	98 (43.8)	43 (51.2)	8 (15.7)	30 (69.8)	17 (37.0)
Middle class	114 (50.9)	35 (41.7)	40 (78.4)	12 (27.9)	27 (58.7)
Rich	12 (5.4)	6 (7.1)	3 (5.9)	1 (2.3)	2 (4.3)
Type of respondent					
Patient	10 (4.5)	4 (4.8)	—	6 (14.0)	—
Caretaker	194 (86.6)	66 (78.6)	51 (100)	35 (81.4)	42 (91.3)
Other	20 (8.9)	14 (16.7)	—	2 (4.7)	4 (8.7)

Abbreviation: NA, not available.

### Diagnosis and Circumstances of Treatment

The most common diagnoses were lymphoma (21.9%), renal tumors (17.4%), retinoblastoma (13.8%), and leukemias (13.8%). More than half of the families (61.3%) had to borrow money or go into debt to pay medical care expenses for CC care (Data Supplement).

### Barriers to Treatment

#### Top Barriers Overall

**FIG 2:**
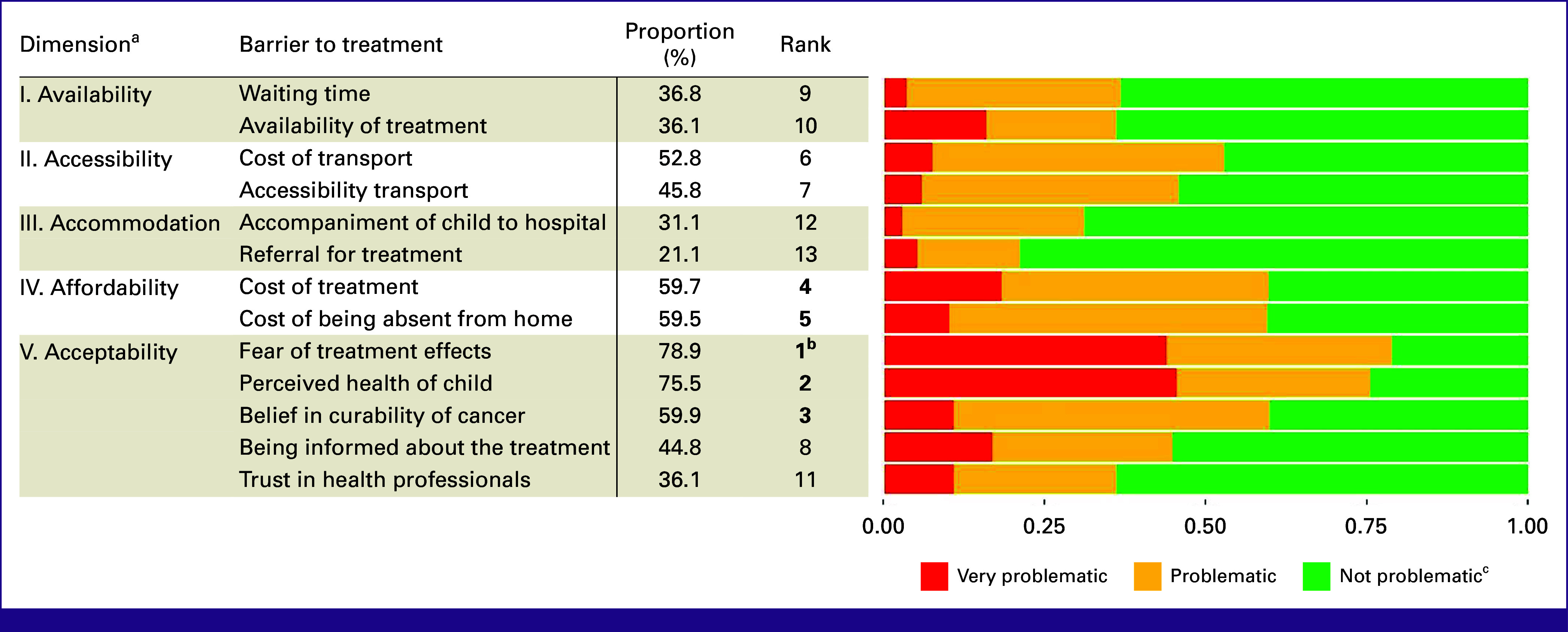
Barriers to treatment. ^a^Barriers grouped according to the 5-A model. ^b^Top five barriers indicated in bold. ^c^Proportion of answers by very problematic, problematic, and not problematic.

When asked about barriers to surgery, radiotherapy, and/or chemotherapy, respondents most frequently mentioned the fear of treatment effects and the perceived (poor) health of the child (78.9% and 75.5%, respectively). Additional barriers frequently named were the (missing) belief in curability of cancer (59.9%), cost of therapy (59.7%), and the cost of being absent from home to accompany the child during their treatment (59.5%; Fig 2).

#### By Treatment Modality and Region

**FIG 3:**
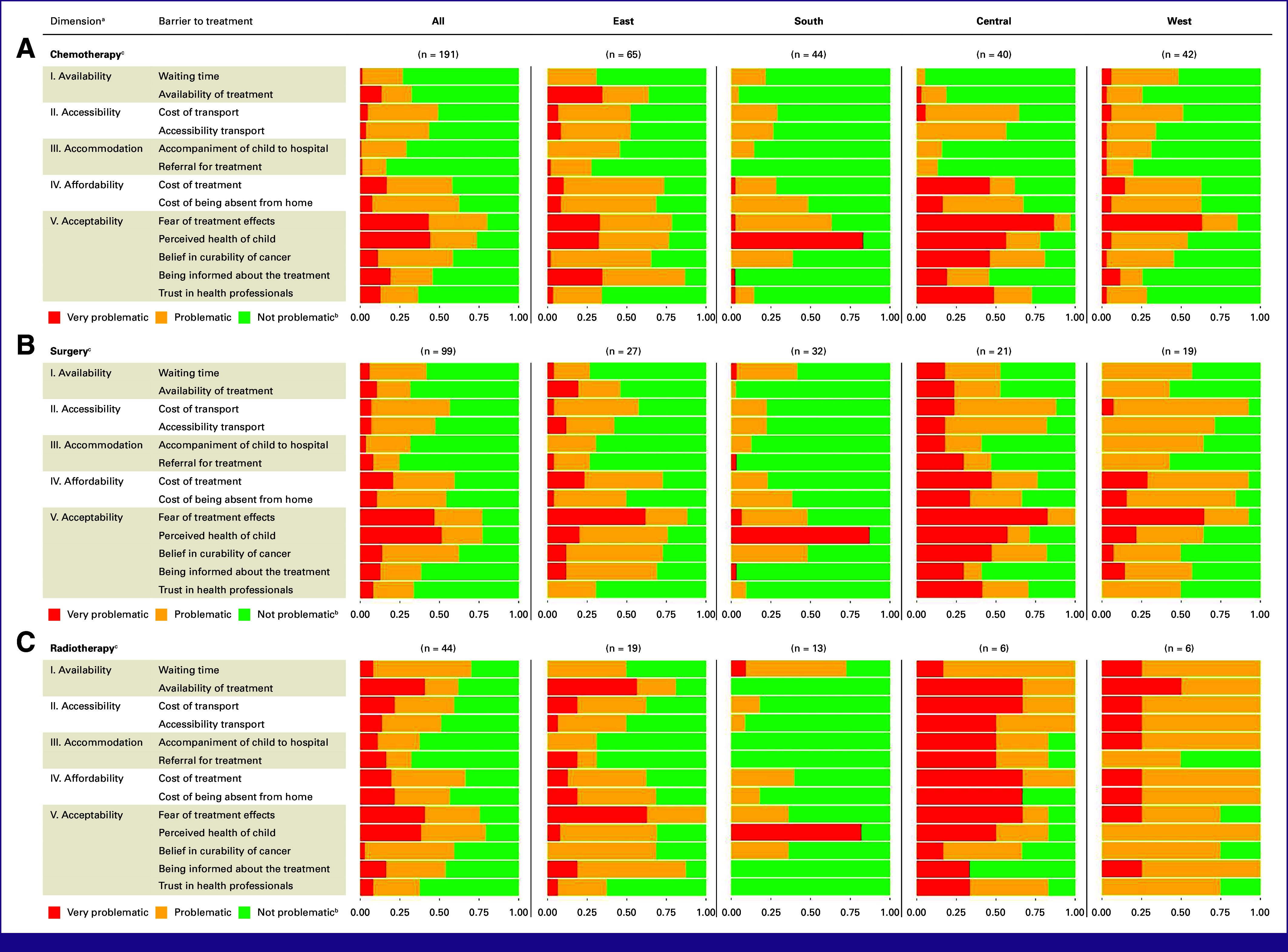
Barriers to treatment stratified by therapy modality and region. ^a^Barriers grouped according to the 5-A model. ^b^Proportion of answers by very problematic, problematic, and not problematic. ^c^Proportion of missing answers by treatment modality: chemotherapy 1.0%; surgery 1.6%; radiotherapy 0.8%.

The fear of treatment effects and the perceived (poor) health of the child were among the most prevalent barriers, irrespective of individual treatment modalities and regions. The (limited) availability of treatment was a considerable barrier for surgery and radiotherapy in all regions except Southern Africa. For chemotherapy, in contrast, (limited) availability was only a frequent barrier in Eastern Africa (63.9% compared with 4.9%-25.7% in other regions). In Eastern Africa, to be (inadequately) informed about the treatment and its circumstances was a common barrier for all three treatment modalities in contrast to other regions. The referral for treatment was in Central and Western Africa a relatively more frequent barrier for surgery and radiotherapy than for chemotherapy (Fig 3; Data Supplement).

#### Multivariable Analysis of Access to Chemotherapy

**FIG 4:**
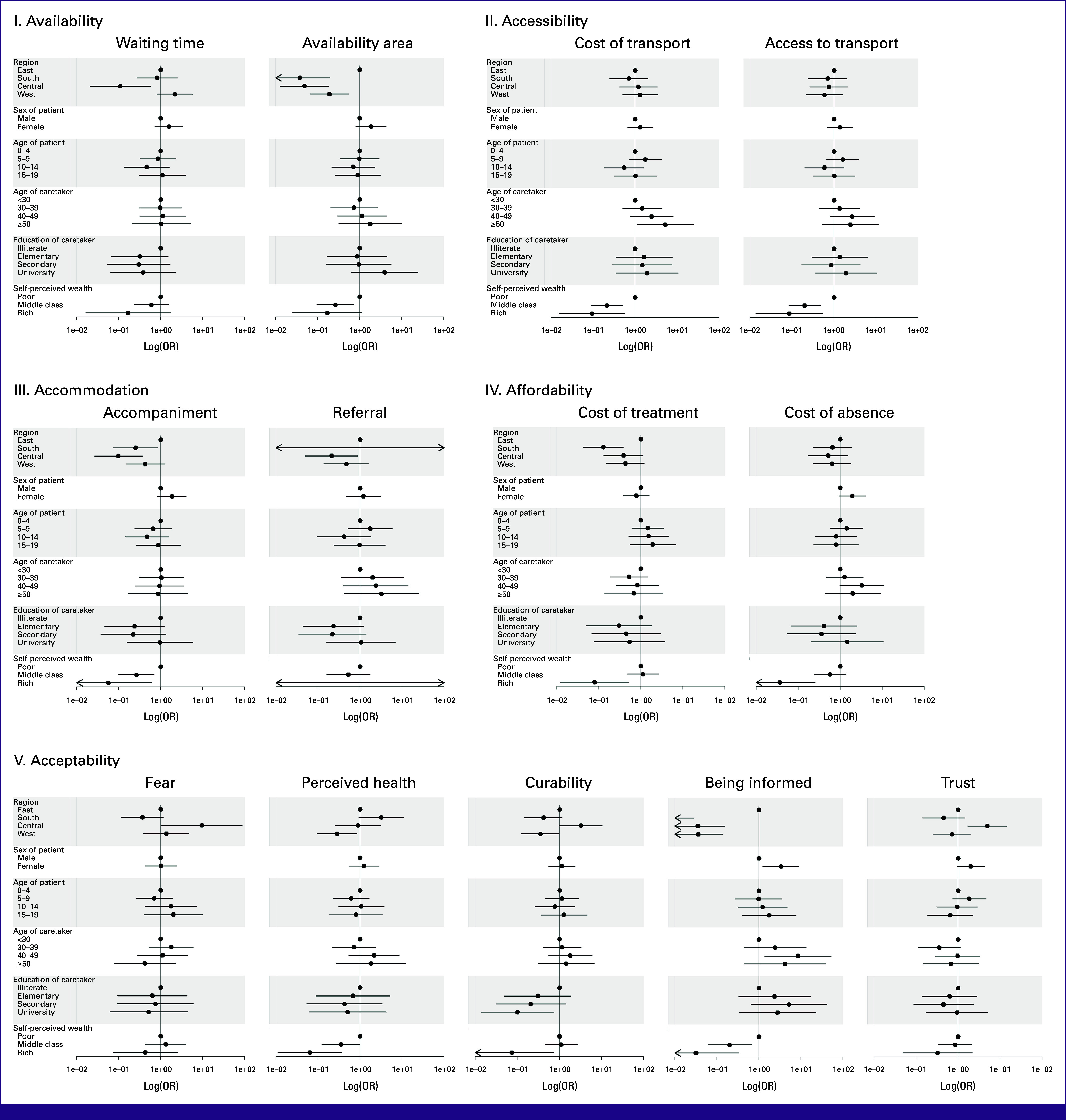
Multivariable analysis of socio-demographic factors association with self-perceived barriers to chemotherapy. Odds ratio (OR) <1 self-perceived barriers less likely reported in the category; OR > 1 self-perceived barriers more likely reported in the category. Barriers grouped according to the 5-A model and results presented as log odds ratio (log(OR)).

Accessing chemotherapy, two factors were relevant for the presence of nearly all barriers: region and self-perceived wealth. The (limited) availability of chemotherapy was less likely a barrier in Southern, Central, and Western Africa (OR, 0.04 [95% CI, 0.01 to 0.19]; OR, 0.05 [95% CI, 0.01 to 0.18]; OR, 0.19 [95% CI, 0.07 to 0.54], respectively) than in Eastern Africa. Similarly, to be (inadequately) informed about the treatment and its circumstances was less likely a barrier in Southern, Central, and Western Africa (OR, 0 [95% CI, 0 to 0.03]; OR, 0.04 [95% CI, 0.01 to 0.15]; OR, 0.04 [95% CI, 0.01 to 0.14], respectively) compared with Eastern Africa. Self-reported association with the middle class or the rich decreased the likelihood of several barriers. To self-identify as rich also decreased the odds of perceiving the (poor) health of the child, the (missing) belief in curability, and to be (inadequately) informed about the treatment as barriers (OR, 0.06 [95% CI, 0.01 to 0.36]; OR, 0.07 [95% CI, 0.01 to 0.73]; OR, 0.03 [95% CI, 0 to 0.33], respectively; Fig 4).

#### Multivariable Analysis of Access to Therapy and Region

Considering associations of region and treatment modality with barriers when accessing therapy, long waiting time and (limited) local availability were more frequently barriers for radiotherapy (OR, 7.53 [95% CI, 3.38 to 16.78]; OR, 11.11 [95% CI, 2.04 to 60.46], respectively) than for chemotherapy, long waiting time especially in Western Africa (OR, 3.13 [95% CI, 1.53 to 6.42]) compared with Eastern Africa. For surgery and radiotherapy, referral was a more common barrier (OR, 2.72 [95% CI, 1.16 to 6.40]; OR, 3.58 [95% CI, 1.21 to 10.61], respectively) than for chemotherapy (Data Supplement).

## DISCUSSION

To our knowledge, this is the first study to compare self-perceived barriers with CC care at a population level in SSA. Our large multinational sample (N = 224) from nine centers allowed us to point out essential opportunities and needs concerning access to CC treatment modalities for further directing cancer control in the region. In line with previous studies, lymphomas and leukemias were common diagnoses among our patients.^12,13^ However, renal tumors and retinoblastoma were more frequent and soft tissue sarcomas less frequent in contrast to previous registry-based studies.

We found that acceptability (beliefs and perceptions between clients and providers) was a major barrier in our study.^7^ Strikingly, the fear of treatment effects and the perceived health of the child were highlighted as the most common barriers to CC care, even when stratified by region and treatment modality. In a hospital-based study of children with a lymphoma diagnosis from Malawi, 15 guardians participated in qualitative interviews and, similarly, named treatment side effects and loss of hope for the child as recurrent reasons for treatment abandonment.^14^ Despite the perceived (poor) health of the child and the fear of treatment effects, in our study, six of 10 respondents named the missing belief in the curability of cancer as a barrier. Previous studies that covered the topic portrayed contradictory results. In a hospital cohort of 99 patients with CC from Kenya, 61% of parents believed CC to be curable, whereas, in a hospital cohort of 82 patients with CC with Burkitt lymphoma from Uganda and Kenya, the majority of guardians was uncertain whether cancer was curable.^6,15^

Previous studies in SSA indicated that little understanding of the child's disease and its treatment was a common challenge for timely and complete CC care.^16^ Concerning access to chemotherapy, our findings suggest that a lack of information about the treatment and its circumstances was less frequently a barrier in Southern, Central, and Western Africa compared with Eastern Africa. Furthermore, barriers as the perceived (poor) health of the child, the (missing) belief in curability, and to be (inadequately) informed about the treatment were less frequent in families that considered themselves as middle class or rich. In a conceptual model from Erdmann et al, it is proposed that a low socioeconomic status affects the coping behavior and the stressors and resources of families of patients with CC negatively.^17^ However, mutual support between affected families seems to help cope with the emotional drain.^18^ In a hospital-based study from Kenya, most parents of patients with CC reported that they shared their thoughts and experiences with other affected parents, and 91% of them recalled that they were provided with emotional support.^18^ To financially support networks of peer support would be one measure to address barriers concerning the acceptability of treatment. Additionally, an increased number of specialized pediatric oncology personnel could contribute to cater for the specific needs of patients with CC and their families.

Chemotherapy, surgery, and radiotherapy are not continuously available for patients with CC in SSA. In our study, long waiting time and (limited) local availability were seven-fold and 11-fold more likely to be barriers for radiotherapy compared with chemotherapy. An analysis from the International Atomic Energy Agency showed that no country in SSA provided sufficient radiotherapy resources to match treatment need.^19,20^ The (limited) availability of chemotherapy was a less common barrier in Southern, Central, and Western Africa in our study compared with Eastern Africa. A report from the SIOP concluded that chemotherapy was continually available in all countries from Eastern Africa (Ethiopia, Tanzania, and Uganda) where the participating registries are located.^20^ However, local availability might have been interrupted intermittently as in a recent analysis on access to essential CC medicines in four East African countries (Kenya, Rwanda, Tanzania, and Uganda).^21^ In a study from Kenya, 26% of parents of patients with CC experienced problems with availability of chemotherapeutics.^22^ Additionally, chemotherapy regimen often consists of several drugs which all need to be consistently available. Ethiopia is one of the countries with the lowest Human Development Index in SSA and is therefore prone to such problems.^23^ Despite the sheer availability of chemotherapeutics, the selection of the necessary regimen also depends on an accurate pathological diagnosis, at best with knowledge of the tumor biology. For example, adapted treatment guidelines for Wilms tumor by the Collaborative Wilms Tumor Africa Project require the tumor (sub)type and the pathology stage.^24^

Some 60% of respondents indicated that the direct cost of treatment and indirect costs due to the caretakers' absence from home were barriers. Those indirect costs are possibly attributable to costs for external child care for siblings or absence from home. In previous studies, caretakers named the cost of care as one of the major challenges after a CC diagnosis.^6,18^ In our study, 61% responded that they went into debt or loaned money for the expenses resulting from the cancer disease. One of the priorities of the United Nations is to provide universal health coverage.^25^ CC needs to be included in national cancer control plans. However, an analysis of 18 cancer control plans in Africa by Weaver et al found that only seven explicitly named CC.^26^ In a hospital-based study from Malawi, 32 caretakers of patients with CC offered their perspective on problems to treatment adherence, and the loss of income or even the loss of the job related to the child's cancer diagnosis altogether was named a problem.^27^ In line with the objective of universal health coverage of the United Nations, to fully fund the costs for CC care for the affected families would be a straightforward mean to reduce the burden of a common barrier to CC care—the costs of treatment.

Accommodation refers to whether the organization of health care services is tailored to the client's needs and is perceived by them in this way. To accompany the child to hospital was, despite the resulting costs, not a notable barrier in our study, in contrast to previous studies that identified household obligations and the impossibility to stay absent from home as challenges to CC care.^10,24^ In our sample, the patients and their families mainly live in urban areas. Possibly, this made it easier to accompany the child to the hospital. Referral for treatment was three-fold more likely a barrier for surgery and four-fold more likely for radiotherapy compared with chemotherapy. The analysis from the SIOP showed that most countries have trained pediatric hemato-oncologists, whereas pediatric oncology surgeons are scarce.^20^ This might complicate referral for surgery. For radiotherapy, the available resources do not match the treatment need as pointed our earlier.^19^

In our study, 50% of respondents perceived both the cost of transport and the accessibility as barriers. Previous studies from SSA identified the cost of transport as one of the major barriers to timely and complete CC treatment. In a hospital cohort of 99 patients with CC from Kenya, 81% of the parents found the transport to hospital to be expensive.^15^ In our (urban) sample, the ability to reach transport to hospital and pay for it was not as frequently a barrier. As opposed to rural settings, the (public) transport system might be accessible for most people in the capital cities and costs lower.

Our study has some limitations. The questionnaire was based on results from previous studies, developed in coordination with Public Health experts and health care professionals from the field and piloted. However, it is not a previously validated instrument, and issues related to reliability and validity cannot be ruled out. Information regarding recommendation and receipt of treatment was based on the assertions of the respondents. However, we moved the perception of the child's disease and its treatment by caretakers to the fore, and thus, clinical data would not have changed our conclusions. The use of terms such as problematic or unproblematic might be subjective and may be understood differently. As the perceptions of the patients with CC and their families were at the core of our interest, we were, however, interested in their subjective views. A potential social-desirability bias may occur regardless. Especially the answers regarding the acceptability of treatment may be affected as the survey was conducted by health care professionals from the registries or linked hospitals. Due to the time between date of diagnosis and data collection, a recall bias could influence results. We included patients with CC diagnosed between 2017 and 2019. For sensitivity analysis, we reviewed completeness of answers and did not notice any differences by year of diagnosis. A small proportion of respondents were contact persons of the families. However, the questionnaire contained several items that requested respondents to confirm that they were well informed about the child's cancer disease and the caretaker. However, their knowledge may be limited in some cases and the recall bias might be more pronounced compared with other respondents. In Harare, only patients with CC up to age 14 years were included. As one of nine centers was affected, we do not believe this to change our overall results and conclusions. Our results are based on a sample of patients from nine PBCRs of over 30 members of the AFCRN and can only represent the state in the respective registries and not all countries in the region. The most common diagnoses slightly differ from results of previous studies which might be influenced by the small number of cases per registry and limit the generalizability.

In conclusion, with numbers of children developing cancer in SSA certain to increase in coming years, the question arises on how to extend CC care with limited resources available. Our study indicates that the caretaker's perceptions of the child's cancer and its treatment are crucial. To expedite the implementation of the SIOP's recommendations on social support for CC treatment in LMIC would help to allay some of the fears of patients with CC and their families.^4^ This may lead to higher rates of completed treatment. Eventually, improved access to therapy and more treatment completion would contribute to meeting the WHO's Global Initiative for Childhood Cancer's survival target.^3^

## Data Availability

The data that support the findings of this study are available from the corresponding author upon reasonable request, which will be evaluated by the AFCRN research committee. Details of the data application process are outlined on the AFRCN website (http://afcrn.org/index.php/research/how-to-apply/76-research-collaborations).
